# Hypoxia-Induced lncRNA-NEAT1 Sustains the Growth of Hepatocellular Carcinoma via Regulation of miR-199a-3p/UCK2

**DOI:** 10.3389/fonc.2020.00998

**Published:** 2020-06-24

**Authors:** Qiangnu Zhang, Qian Cheng, Mengting Xia, Xiaotao Huang, Xiaoyan He, Juan Liao

**Affiliations:** ^1^Department of Hepatobiliary and Pancreas Surgery, The Second Clinical Medical College (Shenzhen People's Hospital), Jinan University, Guangzhou, China; ^2^Integrated Chinese and Western Medicine Postdoctoral Research Station, Jinan University, Guangzhou, China; ^3^Department of Gastroenterology, West China School of Public Health and West China Fourth Hospital, Sichuan University, Chengdu, China; ^4^North Sichuan Medical College, Nanchong, China; ^5^Department of Pathology, The Fifth Affiliated Hospital of Zhengzhou University, Zhengzhou, China

**Keywords:** hepatocellular carcinoma, LncRNA-NEAT1, microRNA, UCK2, hypoxia

## Abstract

**Objective:** The long noncoding RNA (lncRNA) nuclear paraspeckle assembly transcript 1 (NEAT1) has emerged as a novel player in hepatocellular carcinoma (HCC). Hypoxia is a common characteristic of the microenvironment of HCC. This study aimed to investigate whether lncRNA-NEAT1 is induced by hypoxia in HCC, and the mechanism that underlies LncRNA-NEAT1 function.

**Methods:** The expression changes of lncRNA-NEAT1 in HCC cell lines under hypoxic conditions were examined by quantitative reverse transcription-polymerase chain reaction (qRT-PCR). The regulatory effect of HIF-1α on lncRNA-NEAT1 was confirmed with chromatin immunoprecipitation (ChIP) and luciferase reporter assays. The function of lncRNA-NEAT1 on HCC cell growth under hypoxic conditions was determined by CCK-8 assay and flow cytometry. lncRNA -NEAT1 was predicted to serve as a competing endogenous RNA (ceRNA) within microRNA (miRNA)/mRNA axes based on microarray data, public HCC-related datasets and integrative bioinformatics analysis, and the miR-199a-3p/UCK2 axis was selected and validated by qRT-PCR, western blotting, RNA immunoprecipitation, and luciferase reporter analyses. The role of miR-199a-3p/UCK2 in HCC and its functional association with lncRNA-NEAT1 were assessed both *in vitro* and *in vivo*.

**Results:** LncRNA-NEAT1 expression was significantly induced by hypoxia in SNU-182 and HUH7 cells. HIF-1α was shown to regulate lncRNA-NEAT1 transcription. Under hypoxic conditions, lncRNA-NEAT1 maintained the growth of HCC cells and inhibited apoptosis and cell cycle arrest. LncRNA-NEAT1 was predicted to regulate a panel of HCC-associated miRNA-mRNA pairs consisting of 8 miRNAs and 13 mRNAs. LncRNA-NEAT1 was shown to function as a ceRNA of miR-199a-3p/UCK2 both in HCC cells under hypoxic conditions and in an animal model.

**Conclusion:** LncRNA-NEAT1 is a hypoxia-responsive lncRNA in HCC cell lines Insilico evidence suggested that LncRNA-NEAT1 may sustainthe growth of HCC cells by regulating HCC-associated mRNAs that interact with tumor-suppressive miRNAs. The lncRNA-NEAT1/miR-199a-3p/UCK2 pathway may contribute to the progression of HCC cell lines in a hypoxic microenvironment and therefore may represent a novel therapeutic target for HCC.

## Introduction

Hepatocellular carcinoma (HCC) is one of the most commonly diagnosed primary cancers of the liver and the leading cause of cancer-related mortality worldwide, accounting for more than 780,000 deaths in 2018 (1). Although progress has been made in recent years in diagnostic, treatment, and screening technologies, HCC is often diagnosed at advanced stages, and in most cases, advanced HCC patients miss the best surgical window (2). Unfortunately, the effects of chemoradiotherapy, targeted therapy, and immunotherapy against HCC are rather limited, with dismal survival rates for most HCC patients (3). Therefore, there is an urgent need to identify novel targets and molecular markers for the diagnosis and treatment of HCC. Concerted efforts are needed to better understand the pathophysiological mechanisms underlying the development and progression of HCC.

Abnormal vascular networks surrounding solid tumors and the requirement of excess oxygen for rapid growth of cancer cells can lead to hypoxia, which is a common characteristic of the microenvironment of solid tumors (4). At the cellular level, hypoxia induces angiopoiesis, metabolic reprogramming, epithelial–mesenchymal transition, remodeling of extracellular mechanisms, stemness, and immune escape. Clinically, hypoxia contributes to the aggressive clinical characteristics of HCC and resistance to both radiotherapy and chemotherapy (5, 6). Genes, especially those that are regulated by hypoxia inducible factors (HIFs), as well as signal transduction related to the hypoxic microenvironment, have been a common focus over the last decade. Nevertheless, it is still a challenge to treat HCC by targeting hypoxia.

Considerable evidence suggests that hypoxia regulates long non-coding RNA (lncRNAs) that involved in the onset and progression of various cancers. Hypoxia responsive lncRNAs may play clinical roles on patients' progression and prognosis by regulating proliferation, migration, invasion, and therapy resistance of cancer cells (7, 8). For instance, Deng et al. found that lncRNA-BX111887 transcription is induced by HIF-1α in response to hypoxia, which enhances the proliferation and invasion of pancreatic cancer cells (9). LncRNA-MALAT1 expression is also dramatically increased in HCC cells in response to hypoxic conditions, whereas knock-down of MALAT1 counteracts the tumor-promoting effect of hypoxia (10). Thus, elucidation of the roles of lncRNAs under hypoxic conditions is crucial to better understand the onset, features, and poor clinical outcome of HCC.

The lncRNA nuclear paraspeckle assembly transcript 1 (NEAT1) has been reported as a novel player in the onset and progression of HCC. Overexpression of NEAT1 drives HCC progression and has been correlated with the poor prognosis of patients (11, 12). Furthermore, Choudhry et al. demonstrated that lncRNA-NEAT1 activation in response to hypoxia promotes the survival of breast cancer cells (13). However, the activation of lncRNA-NEAT1 in response to hypoxia has not been elucidated in HCC, and lncRNA-NEAT1-regulated downstream pathways are not well-established. Therefore, in the present study, we investigated the response of lncRNA-NEAT1 to hypoxia and revealed its mechanism, which involves transcriptional regulation by HIF-1α. Potential miRNA and mRNA targets of lncRNA-NEAT1 were selected and filtered by an integrative bioinformatics analysis approach that was based on numerous HCC-related datasets. Moreover, candidate miRNA and mRNA targets were validated *in vitro* and *in vivo*.

## Materials and Methods

### Cell Culture and Hypoxic Conditions

HCC cells (SNU-182 and HUH7) were obtained from the American Type Culture Collection (Manassas, VA, USA) and routinely cultured in Roswell Park Memorial Institute 1640 (for SNU-182 cells) or Dulbecco's Modified Eagle's Medium (for HUH7 cells) supplemented with 10% fetal bovine serum, 100 U/mL of penicillin, and 100 mg/mL of streptomycin (Hyclone Laboratories, Inc., South Logan, UT, USA) under an atmosphere of 5% CO_2_ at 37°C. As a model for hypoxia, SNU-182 and HUH7 cells were cultured under an atmosphere of 1% O_2_/5% CO_2_/94% N_2_ for 24 h. we also treated cells with CoCl_2_, a hypoxia mimetic agent (14), to simulated hypoxia. The CoCl_2_ treatment performed under normoxic condition.

### Cell Transfection

Cells were transfected with the plasmid pcDNA3.1-NEAT1 to up-regulate lncRNA-NEAT1 expression, while the plasmid pcDNA3.1-UCK2 was used to overexpress UCK2. The empty plasmid pcDNA3.1 served as a negative transfection control. All plasmid were obtained from GenePharma(Shanghai, China). Small interfering RNA for lncRNA-NEAT1 (siRNA-NEAT1) UCK2 (siRNA-UCK2) and HIF-1α (siRNA- HIF-1α) were used to silence their expression (Genepharma, Shanghai, China). To upregulate candidate miRNAs, miR-mimics were obtained. AllStars Negative Control siRNA was used to transfect cells with siRNA and miR-mimics (Qiagen, Hilden, Germany). Prior to experimentation, the cells were transfected for 24 or 48 h. To obtain stably transfected SNU-182 cells, lentiviral vectors were prepared by Genechem Company (Shanghai, China) and used to deliver lncRNA-NEAT1 (Lv-NEAT1), miR-199a-3p (Lv-miR-199a-3p), and shRNA-UCK2 (Lv-shRNA-UCK2). Successful transfection was confirmed by quantitative reverse transcription-polymerase chain reaction (qRT-PCR) analysis after 48 h (Supplementary Figure 1).

### qRT-PCR Analysis

Total RNA was isolated using TRIzol reagent (Life Technologies Corporation, Carlsbad, CA, USA), quantified, and reverse transcribed into complementary DNA (cDNA) using PrimeScript™ RT Master Mix and the PrimeScript™ RT Reagent Kit (Takara Bio, Inc., Shiga, Japan). Then, the cDNA samples were analyzed using the SYBR® Premix Ex Taq™ II Kit (Takara Bio, Inc., Shiga, Japan) with glyceraldehyde 3-phosphate dehydrogenase (GAPDH) as an internal control. For miRNA analysis, cDNAs were generated and amplified using the Mir-X™ miRNA First-Strand Synthesis and qRT-PCR TB Green® Kit (Takara Bio, Inc., Shiga, Japan). U6 was used as reference for miRNA analysis. The PCR protocol was conducted in accordance with the manufacturer's instructions using the primers shown in Supplementary Table 1.

### Western Blot Analysis

Total protein samples were prepared in radioimmunoprecipitation assay buffer (Beyotime Institute of Biotechnology, Shanghai, China) containing protease inhibitor cocktail. Samples with equal amounts of protein were loaded into the wells of a 12% polyacrylamide gel and separated by electrophoresis. Then, the protein bands were transferred onto nitrocellulose blotting membranes, which were blocked with 5% fat-free milk and incubated with primary antibodies (Abcam, Cambridge, UK) against UCK2 (dilution, 1:1000) and GAPDH (1:2000) for 12 h at 4°C. After incubation with IRDye® secondary antibody (LI-COR Biosciences, Lincoln, NE, USA), the protein bands were imaged using the Odyssey® Infrared Imaging System (LI-COR Biosciences).

### Chromatin Immunoprecipitation (ChIP) Analysis

ChIP analysis was conducted using the ab500 ChIP Assay KIT with anti-HIF-1α antibody (Abcam, Cambridge, UK) in accordance with the manufacturer's instructions. Immunoglobulin G (IgG) served as a control. The resulting DNA fragments were amplified by PCR with the primers listed in Supplementary Table 1. The PCR products were analyzed by electrophoresis.

### RNA Immunoprecipitation (RIP) Assay

RIP analysis was performed using the Imprint® RNA Immunoprecipitation Kit (Sigma-Aldrich Corporation, St. Louis, MO, USA) in accordance with the manufacturer's instructions, with an antibody against argonaute-2 (AGO2, Abcam, Cambridge, UK). IgG served as a control. The purified RNAs were then subjected to qRT-PCR analysis.

### Luciferase Reporter Assay

The 3′ untranslated region of the wild-type (WT) or mutant lncRNA-NEAT1 (or UCK2) sequence was inserted into the psiCHECK-2 luciferase reporter vector (Promega Corporation, Madison, WI, USA). After 48 h of co-transfection with the luciferase reporter vector and miR-199a-3p mimic (or AllStars Negative Control), the cells were lysed, and luciferase activity was measured using the Dual-Luciferase® Reporter Assay System (Promega Corporation). *Renilla* luciferase activity was normalized to firefly luciferase activity. SNU-182 cells or HIF-1α knock-down cells were transfected with luciferase reporter vectors containing the WT or mutant putative hypoxia response element (HRE) sequence (ACGTGC) and then treated with CoCl_2_ for 24 h. A similar luciferase reporter assay was performed to assess the effect of HIF-1α on the promoter of lncRNA-NEAT1.

### Proliferation Analysis

Cell proliferation was assessed using the Cell Counting Kit-8 (CCK-8; Sigma-Aldrich Corporation). In brief, 1 × 10^4^ cells were seeded into the wells of 96-well plates and cultured for 24 h. Then, adherent cells were cultured under normoxic or hypoxic conditions. After 24, 48, or 72 h of culture, the cells were incubated with 10% CCK-8 reagent at 37°C for 1 h. Cell viability was determined by measuring the absorbency at 450 nm. The relative proliferation rate was calculated as the cell viability at 24, 48, or 72 h/cell viability at 0 h. The viability of untreated adherent cells was assessed at 0 h.

### Flow Cytometry Analysis

At 48 h after transfection or 24 h under hypoxic conditions, the cells were harvested and washed with phosphate-buffered saline. The proportion of apoptotic cells was determined by flow cytometry with an Annexin V-FITC Apoptosis Detection Kit (Beyotime Institute of Biotechnology, Shanghai, China). For cell cycle analysis, harvested cells were fixed with 70% cold ethanol for 12 h and then treated with propidium iodide for 30 min. The proportion of apoptotic cells and the cell cycle distribution were measured by flow cytometry using a FACSCalibur™ Flow Cytometer (BD Biosciences, San Jose, CA, USA). Data were analyzed using the FlowJo™ platform for flow cytometry analysis (version 10; FlowJo LLC, Ashland, OR, USA).

### Microarray Analysis

SNU-182 cells were transfected with pcDNA3.1-NEAT1. After 48 h, RNA was collected and analyzed by Agilent Whole human genome Microarray and Human miRNA Microarray, Release 21.0 (Agilent Technologies, Santa Clara, CA, USA). The differentially expressed mRNAs and microRNAs (fold change>1.5 and *P* < 0.05, control *vs*. pcDNA3.1-NEAT1 transfection) were identified using R with Limma package.

### Animal Tumor Model

Twelve BALB/c nude mice, aged 4–6 weeks, were purchased from Biolite Biological Engineering Co., Ltd. (Nanjing, China) and subcutaneously injected with SNU-182 cells stably transfected with Lv-NEAT1, Lv-miR-199a-3p, Lv- shRNA-UCK2, or Lv-control (5 × 10^6^ cells/mouse, *n* = 3 for each group). Tumor diameters were measured every 3 days. The tumor volumes were calculated as 0.5 × (length × width^2^). All mice were sacrificed on day 24, and the tumors were resected and weighed. All animal experiments were performed in accordance with the guidelines of the Research Animal Care Committee of Zhengzhou University (Zhengzhou, Henan, China).

### Statistical Analysis

Statistical analysis was performed with R software (version 3.5.3; https://www.r-project.org/). Normally distributed data are presented as the mean ± standard deviation. Non-normally distributed data are presented as median values. The *t*-test was used to identify significant differences between two sets of normally distributed data, while one-way analysis of variance was used identify differences among multiple groups. Non-normally distributed data were analyzed using the Mann–Whitney *U*-test. The significance of survival data was determined using the log-rank test. A probability (*p*) value of <0.05 was considered statistically significant.

### Bioinformatics Analysis

Liver cancer transcriptome profiling data were downloaded from The Cancer Genome Atlas (TCGA) database (https://www.cancergenome.nih.gov). The expression profile data of one miRNA (GSE36915) and 7 mRNAs (GSE14520, GSE22058, GSE25097, GSE36376, GSE45436, GSE64041, and GSE76427) of HCC patients were retrieved from the Gene Expression Omnibus database (https://www.ncbi.nlm.nih.gov/geo/). Differentially expressed genes and miRNAs were extracted from each dataset with the Limma package of R software (version 3.5.3). The RobustRankAggreg package of R was used for integrated analysis of the expression profile data of the 7 mRNAs. Survival analysis was performed using the “survival” package, and data visualization was performed using the “ggplot2” package of R. The open-source online software (ENCORI v1.0) provided by Encyclopedia of RNA Interactome database (http://starbase.sysu.edu.cn/index.php) was used to predict lncRNA-NEAT1/miRNA interactions. miRWalk 2.0 online software (http://zmf.umm.uni-heidelberg.de/apps/zmf/mirwalk2/) and TargetScan web server (http://www.targetscan.org/vert_72/) were used to predict miRNA-target interactions and the binding sites of candidate miRNAs and mRNAs. Gene ontology enrichment analysis were performed using online tools provided by DAVID Bioinformatics Resources 6.8 (https://david.ncifcrf.gov/). microRNA pathway analyses were conducted using mirPath v.3 (http://snf-515788.vm.okeanos.grnet.gr/).

## Results

### LncRNA-NEAT1 Is Induced by Hypoxia via HIF-1α in HCC Cells

**Figure 1:**
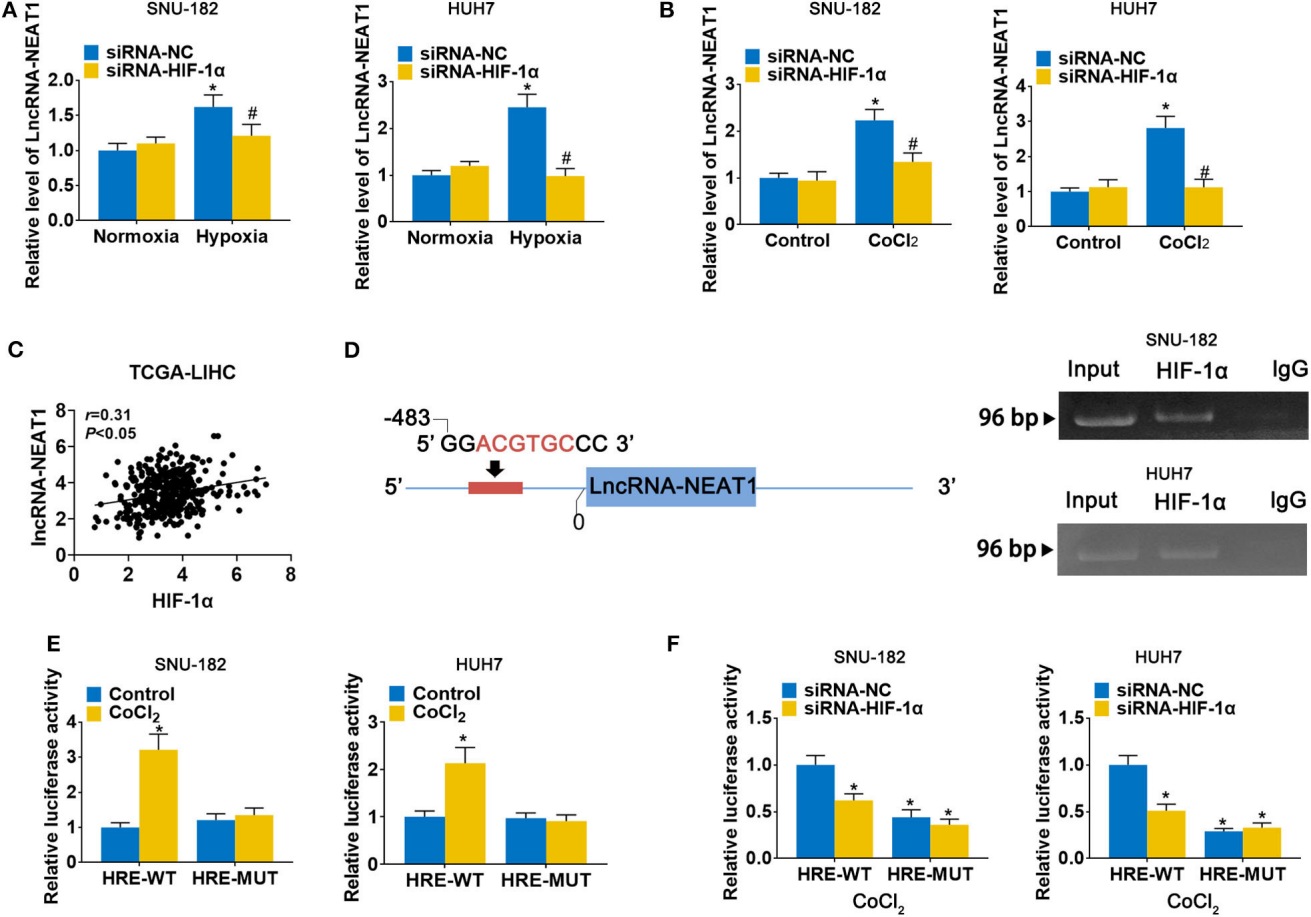
LncRNA-NEAT1 expression is enhanced by hypoxia via transcriptional regulation of HIF-1α in HCC cells. **(A,B)** HIF-1α knock-down and control SNU-182 and HUH7 cells were cultured for 24 h under normoxic vs. hypoxic conditions (*Panel a*) or without and with CoCl_2_ treatment (*Panel b*). Changes in the expression level of lncRNA-NEAT1 were detected by qRT-PCR. **(C)** The correlation of HIF-1α with lnc-NEAT1 in tumor tissues from TCGA-LIHC is shown. **(D)** *Left:* A putative HRE (ACGTGC) was identified in the promoter of lncRNA-NEAT1. *Right:* Binding of HIF-1α to the HRE (ACGTGC) was validated by ChIP assay in SNU-182 and HUH7 cells. HIF-1α antibody or IgG was added to the reaction. DNA fragments were amplified and analyzed by qRT-PCR with specific primers. **(E)** SNU-182 and HUH7 cells were transfected with a luciferase reporter containing the WT or mutant putative HRE (ACGTGC) sequence. Cells were treated with CoCl_2_ for 24 h, where indicated, and relative luciferase activity was detected. **(F)** HIF-1α knock-down SNU-182 and HUH7 cells were transfected with a luciferase reporter containing the WT or mutant putative HRE (ACGTGC) sequence. Cells were treated with CoCl_2_ for 24 h, and relative luciferase activity was detected. In *Panels a and b*, *P < 0.05 compared with normoxia condition or control. ^#^*P* < 0.05 compared with siRNA-negative control (siRNA-NC). In *Panels e and f*, *P < 0.05 compared with normoxia control or siRNA-NC.

To evaluate the response of lncRNA-NEAT1 to hypoxia, we cultured SNU-182 and HUH7 cells under hypoxic conditions (1% O_2_) or treated them with the hypoxia mimetic agent CoCl_2_. As shown in Figures 1A,B, both hypoxic conditions and CoCl_2_ significantly increased lncRNA-NEAT1 expression in SNU-182 cells; however, knock-down of HIF-1α suppressed the response of lncRNA-NEAT1 to hypoxia. Additionally, inspection of the TCGA-LIHC database revealed that HIF-1α expression positively correlates with the lnc-NEAT1 level in HCC tissues (Figure 1C). Therefore, we speculated that transcription of lncRNA-NEAT1 might be regulated by HIF-1α. Inspection of the genomic sequence showed that there is an HRE (ACGTGC) in the lncRNA-NEAT1 3' upstream region that is predicted to bind HIF-1α (Figure 1D, left**)**. We further validated the binding of HIF-1α to the lncRNA-NEAT1 promoter by ChIP assay in SNU-182 and HUH7 cells (Figure 1D, right**)**. Additionally, dual-luciferase reporter assays verified that CoCl_2_ increases the luciferase activity in cells transfected with plasmids containing the WT HRE sequence, but not a mutant sequence (Figure 1E). On the other hand, knock-down of HIF-1α reduced the luciferase activity in CoCl_2_-treated cells for HRE-WT but not HRE-MUT reporter plasmids (Figure 1F). These data indicate that transcriptional upregulation of lncRNA-NEAT1 in HCC cells under hypoxic conditions is mediated by HIF-1α.

### LncRNA-NEAT1 Sustains the Growth of HCC Cells Under Hypoxic Conditions

**Figure 2:**
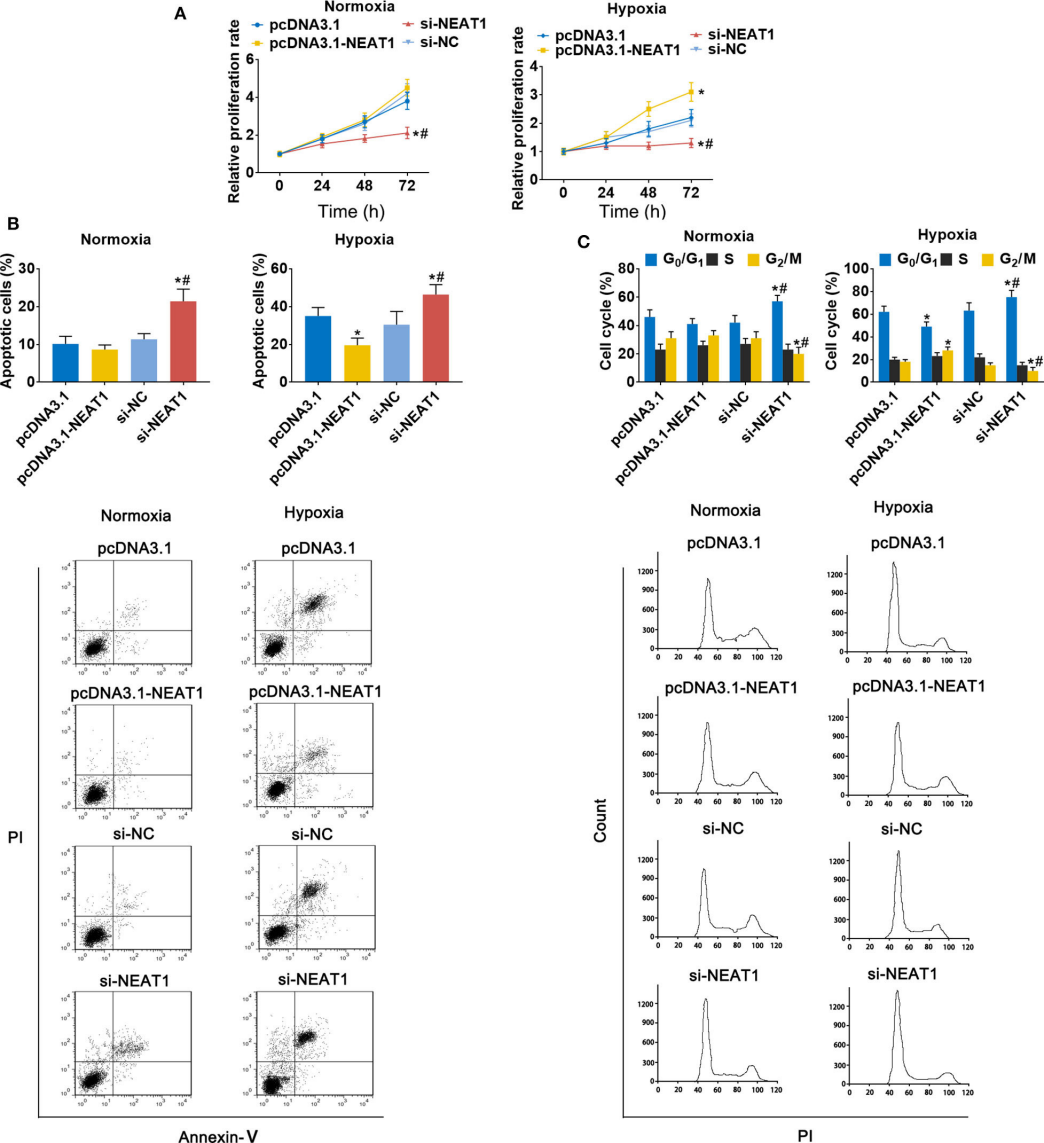
LncRNA-NEAT1 sustains the growth of SNU-182 cells under hypoxic conditions. **(A)** The expression of lncRNA-NEAT1 was modulated by transfection with the pcDNA3.1-NEAT1 vector or siRNA-NEAT1. After transfection for 24 h, cells were cultured under hypoxic or normoxic conditions. Cell viability was detected using CCK-8 assay and used to estimate proliferation. **(B,C)** The roles of lncRNA-NEAT1 on apoptosis (*Panel b*) and the cell cycle (*Panel c*) in SNU-182 cells under hypoxic and normoxic conditions were assessed by flow cytometry. *P < 0.05 compared with transfection negative control. ^#^*P* < 0.05 compared with pcDNA3.1-NEAT1.

To assess the function of lncRNA-NEAT1 in HCC cells in under hypoxic conditions, we evaluated the effect of lncRNA-NEAT1 overexpression and knock-down. Overexpression of lncRNA-NEAT1 had no effect on the viability of SNU-182 cells under normoxic conditions; however, knock-down of lncRNA-NEAT1 inhibited cell viability (Figure 2A, left panel). Furthermore, under hypoxic conditions, cells transfected with pcDNA3.1-NEAT1 had higher proliferation rates, while knock-down of lncRNA-NEAT1 inhibited proliferation (Figure 2A, right panel). Consistently, knock-down of lncRNA-NEAT1 induced apoptosis of SNU-182 cells, while after 24 h of exposure to hypoxic conditions, siRNA-NEAT1 treatment increased the proportion of apoptotic cells, which was reduced by lncRNA-NEAT1 overexpression (Figure 2B). Similarly, overexpression of lncRNA-NEAT1 had no effect on the cell cycle under normoxic conditions. However, lncRNA-NEAT1 knock-down induced G1 arrest after hypoxia treatment for 24 h, and overexpression of lncRNA-NEAT1 antagonized this effect (Figure 2C). These results were confirmed in HUH7 cells (Supplementary Figure 2). Therefore, these findings suggest that lnc-NEAT1 increases the proliferation rate in HCC cells under hypoxic conditions by decreasing the apoptotic rate and promoting G1 arrest.

### Identification of a Panel of HCC-Associated miRNAs and mRNAs That lncRNA-NEAT1 May Regulate as Competing Endogenous RNAs (ceRNAs)

**Figure 3:**
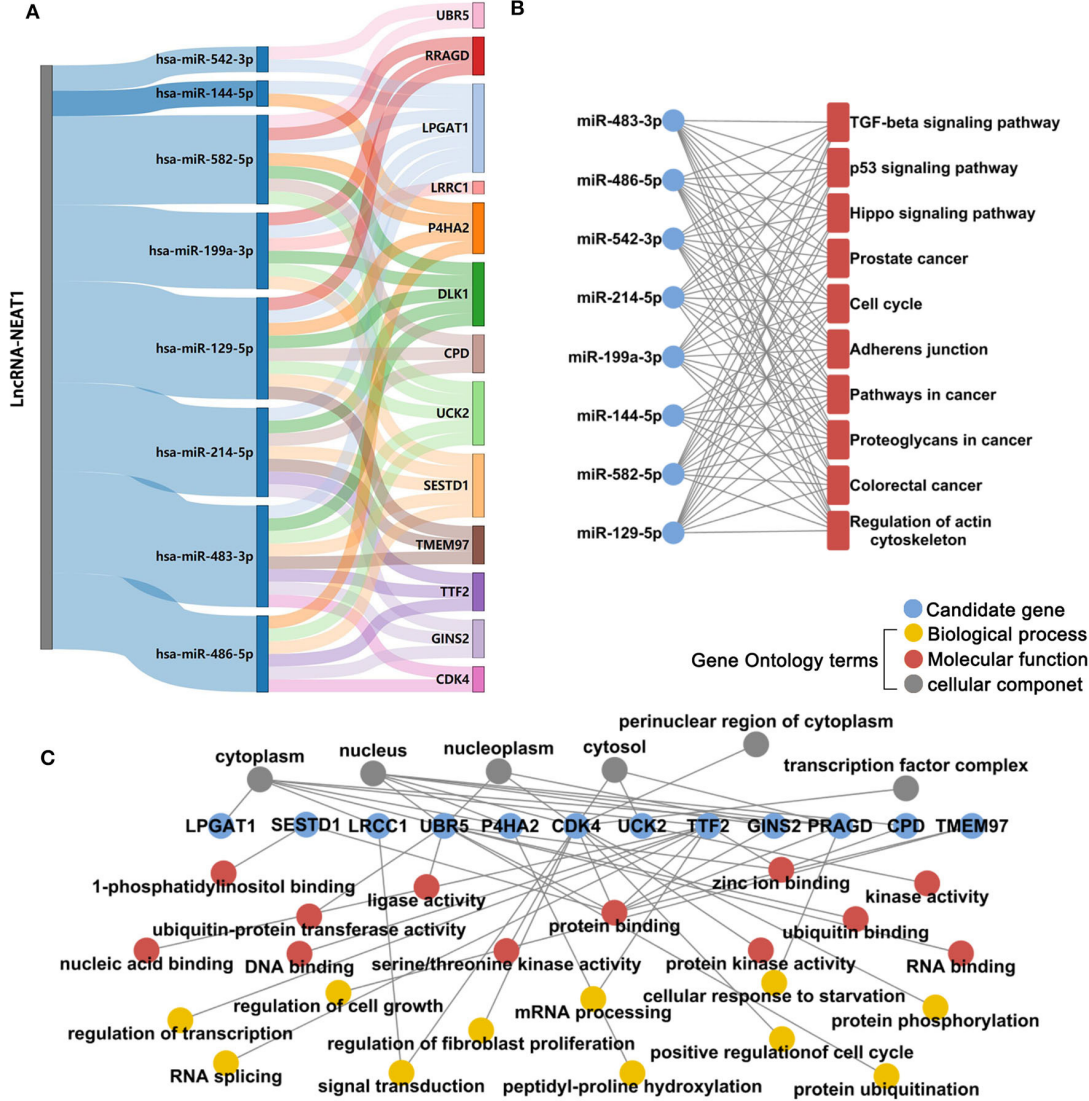
Microarray and integrative bioinformatics analysis of HCC-associated miRNA-mRNA pairs that are potentially regulated by lncRNA-NEAT1. **(A)** Differentially expressed miRNAs and mRNAs in lnc-NEAT1 knock-down SNU-182cells were identified by microarray analysis. According to the ceRNA theory, miRNAs that may be sponged by lncRNA-NEAT1 and their possible target mRNAs were also predicted by bioinformatics analysis and filtered according to their expression patterns in 7 public HCC-related datasets. Candidate miRNA-mRNA pairs that passed each of these filters are shown in the Sankey diagram. The miRNAs that are shown are predicted to act as suppressors in HCC. **(B)** KEGG pathway enrichment analysis for the 8 candidate microRNA's target genes. **(C)** GO enrichment analysis for the 13 candidate genes. GO-biological process terms, -molecular function terms and -cellular component terms are shown.

Next, we investigated the mechanisms underlying the effects of lncRNA-NEAT1 in inducing cell growth under hypoxic conditions. LncRNAs are known to regulate mRNAs by sponging miRNAs as ceRNAs. Firstly, we used microarray analysis to identify the differentially expressed microRNAs and mRNAs (> 1.5-fold with *P* < 0.05, compared with negative transfection control) after pcDNA3.1-NEAT1 transfection in SNU-182 cells. 63 microRNA were significantly down-regulated and 414 mRNA significantly up-regulated after lncRNA-NEAT1 overexpression (Supplementary Tables 2, 3). Next, we built a ceRNA network of potential lncRNA-NEAT1/microRNAs/mRNAs combinations based on the microarray data and bioinformatics predictions (see Supplementary Figure 2 for workflow). CLIP-Seq analysis data derived from online software provided by Encyclopedia of RNA Interactome database were used to identify miRNAs from our microarray analysis containing 7-mer or 8-mer seed matches that are predicted to bind to lncRNA-NEAT1. To further select HCC-associated miRNAs, the miRNAs were filtered with the use of the following criteria: (a) down-regulation by more than 1.5-fold (*P* < 0.05) in HCC tissues according to the GSE36915 dataset; and (b) reported in the literature as cancer suppressors in HCC. Eight candidate miRNAs conforming to these criteria were identified (miR-144-5p, miR-129-5p, miR-199a-3p, miR-214-5p, miR-483-3p, miR-486-5p, miR-542-3p, and miR-582-5p). Next, the targeted mRNAs of these 8 candidate miRNAs were predicted using integrated data from TargetScan and miRWalk 2.0. In total, 780 genes were predicted as target mRNAs of the 8 candidate miRNAs. We compared this list of 780 genes with the set of differentially expressed mRNAs measured in pcDNA3.1-NEAT1 transfected cells. To identify HCC-related genes in the overlapping mRNA list, results were further filtered using the expression profiles results obtained from 7 independent HCC-related public datasets (GSE14520, GSE22058, GSE25097, GSE36376, GSE45436, GSE64041, and GSE76427). An integrated list of differentially expressed genes (HCC-RRA-list) was obtained using the robust rank aggregation (RRA) algorithm with genes that were significantly up- or down-regulated (> 1.2-fold with *P* < 0.05, tumor tissue *vs*. non-tumor tissue) in all 7 HCC-related datasets. Thirteen genes in the overlapping mRNA list were also in the HCC-RRA-list and were up-regulated in HCC tissues. Finally, a potential HCC-associated lncRNA-miRNA-mRNA regulatory flow network was assembled, comprised of lncRNA-NEAT1, 8 candidate miRNAs, and 13 candidate genes (Figure 3A). To better understand the potential biological function of the candidate miRNAs and mRNAs, functional enrichment analysis based on Gene Ontology (GO) and KEGG (Kyoto Encyclopedia of Genes and Genomes) pathway databases was performed. As shown in Figure 3B, the 8 candidate miRNAs were mainly associated with the typical tumor-associated pathways (e.g., cell cycle, p53 signaling pathway, pathway in cancers). The enriched GO-biological process terms, -molecular function terms and -cellular component terms are shown in Figure 3C. Among them, potential terms (e.g., “regulation of cell growth,” “positive regulation of cell cycle,” and “cellular response to starvation”) further support a role for these miRNAs in tumor-related functions.

**Figure 4:**
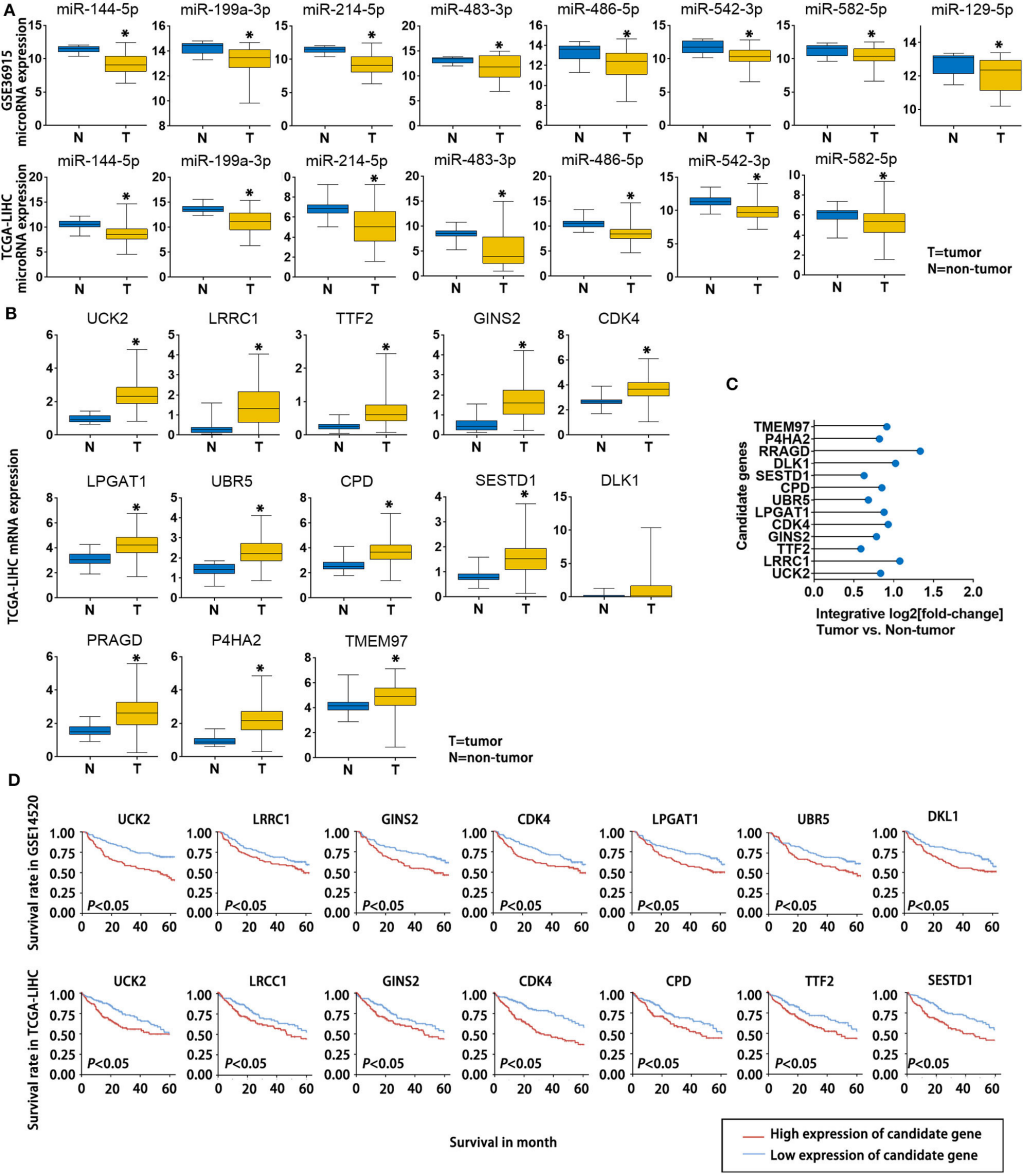
Expression changes and survival relevance of HCC-associated miRNAs and mRNAs that are potentially regulated by lncRNA-NEAT1. **(A)** Expression differences of 8 candidate miRNAs between tumor and non-tumor tissues. Data are from the GSE36915 and TCGA-LIHC datasets. In GSE36915 n(Tumor) = 72, n (non-tumor) = 21. In TCGA-LIHC n(Tumor) = 375, n (non-tumor) = 50. **(B)** Expression difference of 13 candidate mRNAs between tumor and non-tumor tissues in the TCGA-LIHC dataset. n(Tumor) = 375, n (non-tumor) = 50. **(C)** Integrative fold change of 13 candidate mRNAs in tumor tissues as compared with non-tumor tissue in 7 independent HCC datasets obtained from the Gene Expression Omnibus database were calculated by the Robust Rank Aggregation method. **(D)** The associations of the 13 candidate genes with survival are shown using Kaplan–Meier survival curves based on data from the GSE14520 and TCGA-LIHC datasets. In GSE14520, n (High expression of candidate gene) = 123, n (low expression of candidate gene) = 124. In TCGA-LIHC, n (High expression of candidate gene) = 182, n (low expression of candidate gene) = 182.*P < 0.05 compared with normal tissue.

To further verify the potential role of the 8 miRNAs and 13 mRNAs in HCC, we evaluated their expression patterns reported in public databases. The fold changes (tumor tissue *vs*. non-tumor tissue) of the 8 candidate miRNAs in the GSE36915 and The Cancer Genome Atlas Liver Hepatocellular Carcinoma (TCGA-LIHC) datasets are shown in Figure 4A. Except for miR-129-5p, which was not included in the TCGA-LIHC dataset, all of the other candidate miRNAs were consistently down-regulated in HCC tissues in the GSE36915 and TCGA-LIHC datasets. Furthermore, all 13 genes were up-regulated in HCC tumor tissues compared with normal tissue (> 1.5-fold with *P* < 0.05), as confirmed in the TCGA-LIHC dataset (Figure 4B). The integrative fold changes of the 13 candidate genes among the 7 HCC-related public datasets are shown in Figure 4C (tumor tissue *vs*. non-tumor tissue). We further evaluated the potential roles of these genes in HCC prognosis. In the GSE14520 dataset, 7 of 13 candidate genes (UCK2, LRCC1, GINS2, CDK4, LPGAT1, UBR5, and DLK1) are risk factors for poor survival, while according to the TCGA-LIHC dataset, high expression of 7 of the 13 candidate genes (UCK2, LRRC1, TTF2, GINS2, CDK4, CPD, and SESTD1) predict poor survival of HCC patients (Figure 4D). These data indicate that lncRNA-NEAT1 may play a role in the progression of HCC through a panel of HCC-related miRNAs and mRNAs associated with HCC.

### LncRNA-NEAT1 Regulates UCK2 by Sponging miR-199a-3p

**Figure 5:**
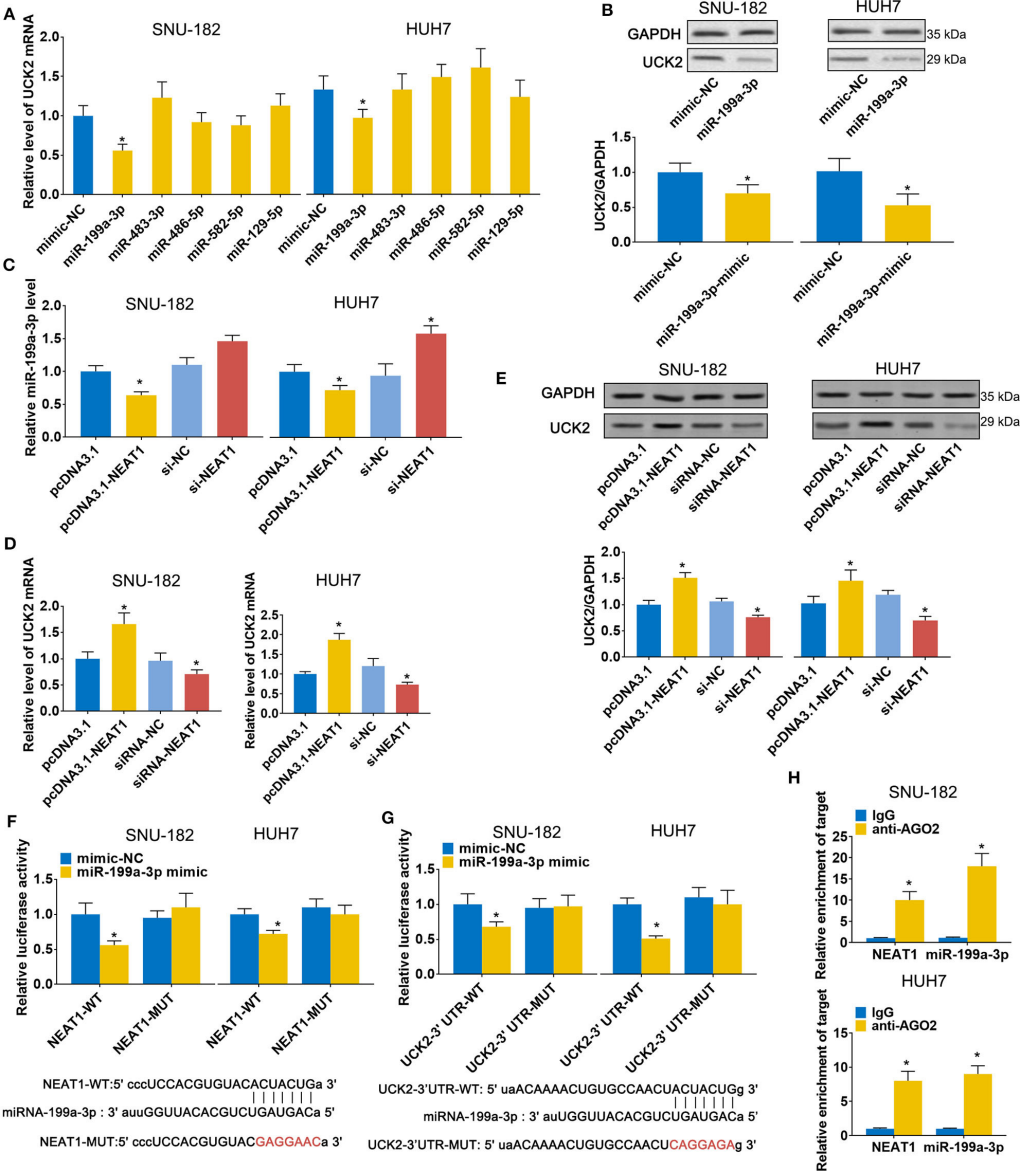
LncRNA-NEAT1 regulates UCK2 by sponging miR-199a-3p in HCC cells. **(A,B)** SNU-182 and HUH7 cells were transfected with miR-mimics of miR-199a-3p, miR-483-3p, miR-486-5p, miR-582-5p, or miR-129-5p. After 48 h, the mRNA and protein expression changes of UCK2 were determined by qRT-PCR (*Panel a*) and western blot analyses (*Panel b*). **(C)** SNU-182 and HUH7 cells were transfected with pcDNA3.1-NEAT1, siRNA-NEAT1, or their respective controls. After 48 h of transfection, changes in miR-199a-3p expression levels were detected by qRT-PCR. **(D,E)** SNU-182 and HUH7 cells were transfected with pcDNA3.1-NEAT1, siRNA-NEAT1, or their respective controls. After 48 h of transfection, changes in UCK2 mRNA (*Panel d*) and protein expression levels (*Panel e*) were determined by qRT-PCR and western blot analyses. **(F,G)** The binding of lncRNA**-**NEAT1 to miR-199a-3p (*Panel f*) and miR-199a-3p to the 3' untranslated region of UCK2 (*Panel g*) were verified by luciferase reporter assay. Wild-type and Mutant sequences of NEAT1 or UCK2 3' UTR are shown at the bottom. **(H)** RIP assay was performed to further confirm whether lncRNA-NEAT1 regulates miR-199a-3p as a ceRNA. Cell lysates collected from SNU-182 and HUH7 cells were incubated with antibodies against AGO2 or IgG. Enrichment of lncRNA-NEAT1 and miR-199a-3p in purified RNA was detected by qRT-PCR. In *Panels a-g*, *P < 0.05 compared with transfection negative control. In *Panel h*, *P < 0.05 compared with lgG control.

Next, we sought to validate the predicted candidate lncRNA-NEAT1-miRNA-mRNA regulatory patterns. Among the 4 genes (UCK2, LRCC1, GINS2, and CDK4) that were identified to be associated with poor survival of HCC patients in both the GSE14520 and TCGA-LIHC datasets, UCK2 had the greatest number of predicted interactions with NEAT1-targeted miRNAs (miR-199a-3p, miR-483-3p, miR-486-5p, miR-582-5p, and miR-129-5p) and, thus, can be regarded as a potential hub mediator. Furthermore, UCK2 was found to be an impressive risk factor for survival (hazard ratio = 2.2, *P* < 0.01 in the GSE14520 dataset; hazard ratio = 2.1, *P* < 0.01 in the TCGA-LIHC dataset). Therefore, we sought to further validate the functional interaction of UCK2, along with miR-199a-3p, miR-483-3p, miR-486-5p, miR-582-5p, and miR-129-5p, with lncRNA-NEAT1. As shown in Figure 5A, only miR-199a-3p mimic influenced UCK2 mRNA expression in SNU-182 and HUH7 cells. The inhibitory effect of miR-199a-3p on UCK2 was confirmed by western blotting (Figure 5B). Furthermore, overexpression of lncRNA-NEAT1 was found to suppress the expression of miR-199a-3p (Figure 5C) and enhance UCK2 mRNA and protein expression (Figures 5D,E), while lncRNA-NEAT1 knock-down had the opposite effect. The role of miR-199a-3p in mediating the effect of lncRNA-NEAT1 on UCK2 expression was further verified by luciferase reporter assay results, which demonstrated that miR-199a-3p decreases luciferase activity in cells transfected with reporter plasmids containing either the NEAT1-WT or UCK2-WT sequences that are predicted to be binding sites for miR-199a-3p, but not corresponding mutant sequences (Figures 5F,G). In addition, the results of RIP assays demonstrated that lncRNA-NEAT1 and miR-199a-3p were both contained in complexes that were pulled down with AGO2 antibody (Figure 5H), which suggests that lncRNA-NEAT1 resides within RNA-induced silencing complexes that are involved in miRNA processing. Taken together, these data indicate that lncRNA-NEAT1 regulates UCK2 by sponging miR-199a-3p as a ceRNA.

### LncRNA-NEAT1 Sustains the Growth of HCC Cells Under Hypoxic Conditions via the Regulation of miR-199a-3p/UCK2

**Figure 6:**
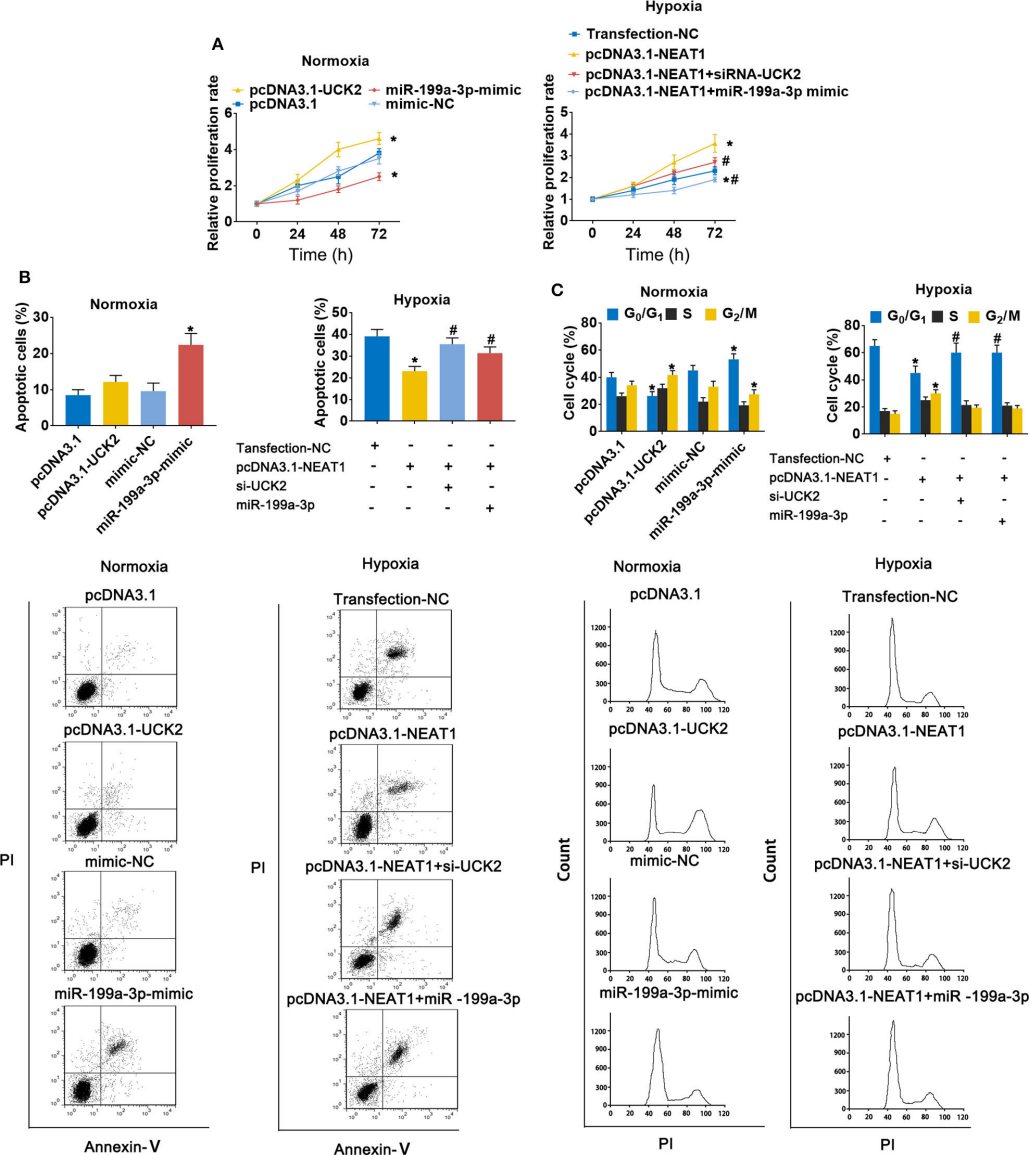
LncRNA-NEAT1 sustains the growth of SNU-182 cells under hypoxic conditions by regulating miR-199a-3p/UCK2. **(A)** The expression levels of miR-199a-3p and UCK2 in SNU-182 were up-regulated by transfection under normoxic conditions; or miR-199a-3p or siRNA-UCK2 were co-transfected with pcDNA3.1-NEAT1 in SNU-182 cells under hypoxic conditions. The cell proliferation changes were determined by CCK-8 assay. **(B,C)** The effects of miR-199a-3p and UCK2 on apoptosis (*Panel b*) and the cycle (*Panel c*) of SNU-182 cells were determined by flow cytometry. *P < 0.05 compared with transfection negative control. ^#^*P* < 0.05 compared with pcDNA3.1-NEAT1.

To further evaluate the role of the lnc-NEAT1/miR-199a-3p/UCK2 axis in lnc-NEAT-1-mediated growth promotion, we repeated the MTT assays in cells transfect with UCK2 overexpression vector or miR-199a-3p mimic. Under normoxic conditions, UCK2 overexpression significantly enhanced cell proliferation, while miR-199a-3p overexpression produced opposite results. Furthermore, under hypoxic condition, UCK2 knock-down or miR-199a-3p overexpression significantly neutralized the sustaining effect of lncRNA-NEAT1 on cell proliferation (Figure 6A). The converse trend was observed in our evaluation of cell apoptosis, for which miR-199a-3p-mimic caused an increase in apoptosis under normoxic conditions, and si-UCK2 and miR-199a-3p neutralized the reduction in apoptosis levels mediated by lncRNA-NEAT1 overexpression under hypoxic conditions (Figure 6B). A similar trend was also observed for cell cycle arrest (Figure 6C), and these results were confirmed in HUH7 cells (Supplementary Figure 4), which suggests that UCK2 and miR-199a-3p have critical roles in lncRNA-NEAT1-induced HCC cell promotion under conditions of hypoxia.

### LncRNA-NEAT1 Promotes HCC Tumor Growth Through miR-199a-3p/UCK2 *in vivo*

**Figure 7:**
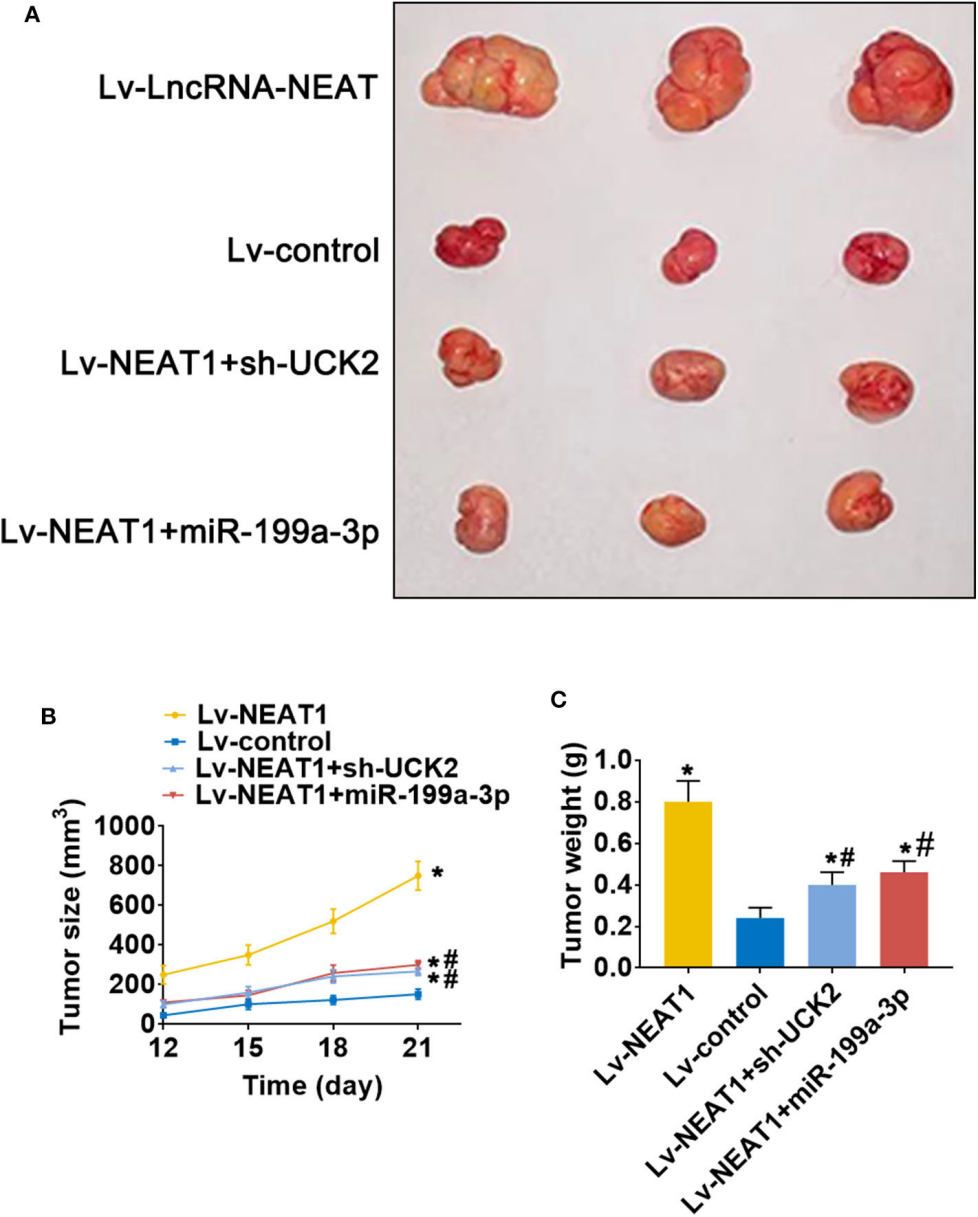
LncRNA-NEAT1 promotes HCC tumor growth through miR-199a-3p/UCK2 *in vivo*. **(A)** SNU-182 cells transfected with Lv-NEAT1, Lv-NEAT1 + Lv-miR-199a-3p, or Lv-NEAT1 + Lv-siRNA-UCK2 were subcutaneously injected into nude mice. After 3 weeks, the tumor formation was examined. **(B)** Tumor diameters were measured every 3 days. **(C)** Tumor weights were measured on day 21 post injection. *P < 0.05 compared with transfection negative control. ^#^*P* < 0.05 compared with Lv-NEAT1.

To determine whether lnc-NEAT contributes to HCC by a similar mechanism *in vivo*, we evaluated the effect of lncRNA-NEAT1, sh-UCK2 and miR-199a-3p expression in xenografted mouse tumors from SNU-182 cells. LncRNA-NEAT1 promoted the development of xenografted tumors (Figure 7A), which was evidenced by larger tumor volumes (Figure 7B) and higher tumor weights (Figure 7C). However, co-transfection of either miR-199a-3p mimic or sh-UCK2 inhibited these promotive effects of lncRNA-NEAT1. Thus, these findings support the role of the lnc-NEAT1/miR-199a-3p/UCK2 axis in HCC tumor growth *in vivo*.

## Discussion

Hypoxia-regulated lncRNAs play pivotal roles in the development of various cancers, including HCC, gastric cancer, and pancreatic cancer, by regulating cellular proliferation, invasion, metastasis, metabolism, and autophagy (15). As an example, Zhao et al. found that lncRNA-MALAT1 is significantly overexpressed in HCC cells under hypoxic conditions, whereas knock-down of MALAT1 weakened the promotive effect of hypoxia on cellular proliferation, migration, and invasion (10). Zhang et al. reported that lncRNA-PCGEM1 is induced in GC cells under hypoxic conditions and acts as an oncogenic factor (16), while up-regulation of lncRNA-BX111 in response to hypoxia promotes metastasis and progression of pancreatic cancer (9). In the present study, we confirmed the response of lncRNA-NEAT1 to hypoxia and demonstrated that lncRNA-NEAT1 is transcriptionally regulated by HIF-1α in HCC cells. Integrated analysis of public HCC-related datasets was performed to select a group of HCC-associated miRNA-mRNA pairs that could potentially be modulated by lncRNA-NEAT1 in a ceRNA-related manner. Moreover, the regulatory effects of lncRNA-NEAT1 on the miR-199a-3p/UCK2 axis in HCC were validated both *in vitro* and *in vivo*.

LncRNA-NEAT1 has been established as a target for the diagnosis and treatment of various solid tumors. Elevated expression of lncRNA-NEAT1 drives tumor initiation and progression by regulating cellular growth, migration, invasiveness, epithelial-to-mesenchymal transition, and stemness (17, 18). Furthermore, lncRNA-NEAT1 has been reported to affect the sensitivity to sorafenib and radiotherapy, as well as immune escape in HCC *in vitro*. Consistent with this role, aberrant expression of lncRNA-NEAT1 has been demonstrated in HCC and is associated with poor survival of HCC patients (11, 19–21). Liu et al. demonstrated that high expression of lncRNA-NEAT1 in a Chinese population is an independent risk factor for poor survival of patients with HCC (22). Furthermore, Ling et al. evaluated the expression level and clinical relevance of lncRNA-NEAT1 in HCC based on data from TCGA-LIHC and other HCC datasets from the Oncomine database, and found that lncRNA-NEAT1 is consistently up-regulated in HCC tumor tissues, though in the TCGA-LIHC dataset, lncRNA-NEAT1 was not significantly associated with overall patient survival but was significantly correlated to distant metastasis. Combined with data from *in vitro* experiments showing that knock-down of lncRNA-NEAT1 inhibits proliferation and induces apoptosis, Ling et al. suggested that lncRNA-NEAT1 promotes deterioration in HCC (23). In the present study, we focused more on the up- and downstream regulatory mechanisms of lncRNA-NEAT1 in HCC rather, than on its tumor-promoting role under conventional conditions, and our results, therefore, may be increase the mechanistic understanding of lncRNA-NEAT1 in each of these prior investigations.

In general, the abnormal expression of lncRNA-NEAT1 in cancer cells is known to be caused by genetic alterations, transcription factors, DNA methylation, miRNAs, and RNA-binding proteins (24). Emerging evidence indicates that hypoxia can modulate the expression of lncRNAs, including lncRNA-NEAT1. Up-regulation of lncRNA-NEAT1 has been found in breast cancer cells under hypoxic conditions (13) and in cardiomyocytes (25), further indicating that lncRNA-NEAT1 may be a hypoxia-responsive lncRNA. However, the response of lncRNA-NEAT1 to hypoxia in HCC has not been well-elucidated. We investigated changes in lncRNA-NEAT1 expression levels in HCC cells under hypoxic conditions (1% O_2_) or treatment with the hypoxia mimetic CoCl_2_. Both the 3.7-kb poly-adenylated NEAT1-1 and the 23-kb non-adenylated NEAT1-2 are upregulated by hypoxia (13). However, NEAT1_1 is a highly conserved and abundant poly-adenylated transcript, which is much more abundant than the longer NEAT1_2 isoform (26). Hence in our study we focused on NEAT1_1. Our data verify that lncRNA-NEAT1 is induced by hypoxia in HCC cells. HIF-1α has been established as a predominant transcriptional regulator in response to hypoxia with the ability to binding to HREs and enhance expression of target genes, including lncRNAs (27). By accessing the JASPAR database, we identified a potential putative HIF-1α-related HRE (5'-ACGTGC-3') located in the promoter of lncRNA-NEAT1. Knock-down of HIF-1α eliminated the response of lncRNA-NEAT1 to hypoxia. Furthermore, the results of the ChIP and luciferase reporter assays supported the binding of HIF-1α to the promoter of lncRNA-NEAT1, suggesting that lncRNA-NEAT1 is transcriptionally induced by HIF-1α. These results provide evidence for the role of HIF-1α and the HRE in the lncRNA-NEAT1 promoter as a mechanism that regulates lncRNA-NEAT1 expression under conditions of hypoxia.

We further demonstrated that overexpression of lncRNA-NEAT1 does not promote proliferation of HCC cells under normoxic conditions, possibly because the overactive proliferative properties of tumor cells under normal conditions may obscure the effect of lncRNA-NEAT1 overexpression by the “ceiling effect.” In addition, the endogenous lncRNA–NEAT1 in HUH7 and SNU-182 cells may be redundant, so in the absence of stress (such as hypoxia), overexpression of lncRNA-NEAT1 cannot promote cell proliferation without limitation. Under hypoxic conditions, the proliferation of HCC cells is inhibited, which provides an opportunity for lncRNA-NEAT1 to function.

As expected, knock-down of lncRNA-NEAT1 inhibited the growth of HCC cells, which was evident both under normoxic and hypoxic conditions. Moreover, acute hypoxia inhibited growth and promoted apoptosis and cell cycle arrest of HCC cells, and HCC cells overexpressing lncRNA-NEAT1 grew relatively faster with less apoptosis and G1 phase arrest. Therefore, these results suggest that increased lncRNA-NEAT1 levels sustain the growth of HCC cells under hypoxic conditions.

LncRNAs regulate gene expression in cancers through distinct mechanisms. For instance, lncRNAs may regulate target genes by specific recruitment of transcriptional activators or suppressors; by acting as decoys that bind to and block transcription factors from target genes; or by recruiting chromatin-remodeling complexes as scaffolding proteins, thereby affecting target genes (28). In tumor biology, lncRNAs primarily serve as ceRNAs that sponge tumor–promotive or tumor-suppressive miRNAs. Sponged miRNAs lose their regulatory effect on target mRNAs, which ultimately influences tumor progression (29). Thus, we employed microarray analysis and a series of advanced online bioinformatics tools to identify potential miRNA-mRNA pairs that may interact with lncRNA-NEAT1 according to a ceRNA mechanism. The candidate miRNA-mRNA pairs were filtered according to their reported suppressive functions and predicted interactions with tumor-related genes, as well as by their down-regulated expression patterns in HCC. To obtain more evidence for the roles of candidate miRNAs in HCC, we performed pathway annotation analysis of predicted miRNA targets based on the KEGG database. The candidate miRNAs were determined to be involved in multiple tumor-related pathways. To filter HCC-related mRNA targets of lncRNA-NEAT1-miRNAs, 7 independent datasets from HCC patients with different backgrounds were used for integrated analysis using the RRA method, which strengthened the evidence. Eight candidate mRNAs were up-regulated in HCC tissues in the 7 datasets. To better explore the potential biological functions of the candidate mRNAs, GO enrichment analysis was performed. Enriched GO-terms, such as “regulation of cell growth” and “positive regulation of cell cycle,” provided a potential explain of how lncRNA-NEAT1 may sustain growth of HCC cells by regulating the candidate mRNAs identified in our study. Though we could not perform a detailed analysis of all candidate miRNA-mRNA pairs in the present study, we selected UCK2, a hub target gene and impressive prognosis risk factor of HCC, for confirmation. The miRNAs that potentially regulate UCK2, including miR-199a-3p, miR-483-3p, miR-486-5p, miR-582-5p, and miR-129-5p, were considered. However, definite interactions were verified only for the lncRNA-NEAT1-miR-199a-3p-UCK2 axis. The results of ChIP and luciferase analyses confirmed the binding of lncRNA-NEAT1/miR-199a-3p and miR-199a-3p/UCK2, thus providing a downstream mechanism that may regulate lncRNA-NEAT1 function.

Previous studies have reported that miR-199a-3p acts as a tumor suppressor via various mechanisms in HCC. For instance, miR-199a-3p inhibits tumor growth in an animal model of HCC by modulating the mTOR pathway (30). Giovannini et al. suggested that miR-199a-3p down-regulation is a common characteristic of HCC and that miR-199a-3p regulates E-cadherin expression through Notch1 (31), Jia et al. reported that miR-199a-3p represses tumorigenesis in HCC by targeting HIF-1α (32). Fornati et al. showed that miR-199a-3p modulates the cell cycle of HCC cells and sensitizes these cells to hypoxia-induced apoptosis (33). In the present study, the suppressive role of miR-199a-3p was confirmed and expression changes of lncRNA-NEAT1 were shown to induce alteration of miR-199a-3p in HCC cells. Furthermore, luciferase reporter and RIP assay demonstrated that lncRNA-NEAT1 sponges miR-199a-3p, which is consistent with our other bioinformatics and experimental data.

Functionally, UCK2 is a pyrimidine ribonucleotide kinase that catalyzes phosphorylation of uridine to uridine monophosphate and cytidine to cytidine monophosphate. Overexpression of UCK2 is regarded as an indicator of unfavorable prognosis in various cancers, including HCC, pancreatic cancer, breast cancer, and lung cancer (34–37). However, few studies have revealed the detailed mechanisms underlying the regulation of UCK2. Zhou et al. found that UCK2 promotes metastasis via up-regulation of MMP2/9 expression and activation of STAT3 signaling (38). The upstream mechanisms of UCK2, especially those involved with lncRNA/miRNA, had not been clarified prior to this study. Therefore, we confirmed the growth-promotive effect of UCK2 in HCC cells and demonstrated that UCK2 is regulated by lncRNA-NEAT1/miR-199a-3p. Most importantly, lncRNA-NEAT1 was shown to function under hypoxic conditions partly through miR-199a-3p/UCK2. Moreover, an animal model was used to further explore the role and regulatory relationship of lncRNA-NEAT1/miR-199a-3p/UCK2. As a limitation of this study, some miRNAs and mRNAs that may be also controlled by lncRNA-NEAT1 were not validated so that we could focus our efforts on validating the lncRNA-NEAT1/miR-199a-3p/UCK2 axis. These miRNAs or mRNAs should be investigated in future studies.

In conclusion, we identified lncRNA-NEAT1 as a hypoxia-responsive lncRNA in HCC cell lines *in vitro*. Based on *in silico* data, we suggested that lncRNA-NEAT1 sustains the growth of HCC cells under hypoxic conditions. LncRNA-NEAT1 may regulate a panel of HCC-associated mRNAs by interacting with tumor-suppressive miRNAs in HCC. The roles of lncRNA-NEAT1/miR-199a-3p/UCK2 were validated HUH7 and SNU-182 cells, which indicated that lncRNA-NEAT1 and its downstream miRNAs/mRNAs may contribute to the progression of HCC cells in hypoxic microenvironments and, therefore, are potential targets for novel therapeutic strategies for HCC.

## Data Availability Statement

The datasets analyzed for this study can be found in the Cancer Genome Atlas (TCGA) database (https://www.cancergenome.nih.gov). The expression profile data of one miRNA (GSE36915) and 7 mRNAs (GSE14520, GSE22058, GSE25097, GSE36376, GSE45436, GSE64041, and GSE76427) of HCC patients were retrieved from the Gene Expression Omnibus database (https://www.ncbi.nlm.nih.gov/geo/).

## Ethics Statement

The animal study was reviewed and approved by Research Animal Care Committee Zhengzhou University.

## Author Contributions

QZ and JL: study design. QC, MX, XHe, and XHu: acquisition of data. QZ and QC: analysis and interpretation of data. QZ and QC: drafting of the manuscript. QZ and QC statistical analysis. JL: funding and study supervision. All authors read and approved the final manuscript.

## Conflict of Interest

The authors declare that the research was conducted in the absence of any commercial or financial relationships that could be construed as a potential conflict of interest.
