# Supplementary material for: Hypoxia-Induced lncRNA-NEAT1 Sustains the Growth of Hepatocellular Carcinoma via Regulation of miR-199a-3p/UCK2
Source: Front Oncol. 2020 Jun 24;10:998. doi: 10.3389/fonc.2020.00998 (PMC7327087; doi:10.3389/fonc.2020.00998)
Supplement: Supplementary file 1 [file Data_Sheet_1.DOCX]

**Supplementary table 1 Primers sequences** **used in this study**

| Targets | sequences | |
| --- | --- | --- |
|  | Forward:5’-3’ | Reverse:5’-3’ |
| LncRNA-NEAT1 | CTTCCTCCCTTTAACTTATCCATTCAC | CTCTTCCTCCACCATTACCAACAATAC |
| UCK2 | CTAGCGGCAAGTCTTCCGTG | TGCTGCACGATGAGGTTGAT |
| GAPDH | GTCTCCTCTGACTTCAACAGCG | ACCACCCTGTTGCTGTAGCCAA |
| U6 | CTCGCTTCGGCAGCACA | AACGCTTCACGAATTTGCGT |
| microRNA-199a-3p | acagTagTcTgcacaTTggTTa | mRQ 3’ Primer in kit |
| microRNA-483a-3p | TcacTccTcTccTcccgTcTT | mRQ 3’ Primer in kit |
| microRNA-486a-3p | cggggcagcTcagTacaggaT | mRQ 3’ Primer in kit |
| microRNA-582-5p | TTacagTTgTTcaaccagTTacT | mRQ 3’ Primer in kit |
| microRNA-129a-5p | cTTTTTgcggTcTgggcTTgc | mRQ 3’ Primer in kit |
| Primers in ChIp assay | AACCACCGCCCGAAAGTC | CCGGCAGGACATCTGGAAAA |

**Supplementary table 2 Significantly down-regulated microRNAs in pcDNA3.1-NEAT1 transfected SNU-182 cells**

| hsa-miR-942-3p | hsa-miR-302c-3p | hsa-miR-10b-3p | hsa-miR-671-5p |
| --- | --- | --- | --- |
| hsa-miR-3138 | hsa-miR-4480 | hsa-miR-129-5p | hsa-miR-519e-5p |
| hsa-miR-515-3p | hsa-miR-199a-3p | hsa-miR-95-3p | hsa-miR-582-5p |
| hsa-miR-640 | hsa-miR-143-3p | hsa-miR-532-3p |  |
| hsa-miR-214-5p | hsa-miR-483-3p | hsa-miR-486-5p |  |
| hsa-miR-4266 | hsa-miR-301a-5p | hsa-miR-204-3p |  |
| hsa-miR-514b-3p | hsa-miR-34a-5p | hsa-miR-7978 |  |
| hsa-miR-6507-5p | hsa-miR-7161-5p | hsa-miR-1301-5p |  |
| hsa-miR-329-5p | hsa-miR-6077 | hsa-miR-128-3p |  |
| hsa-miR-4429 | hsa-miR-6890-3p | hsa-miR-659-5p |  |
| hsa-miR-519d-5p | hsa-miR-194-3p | hsa-miR-6784-5p |  |
| hsa-miR-106a-5p | hsa-miR-139-5p | hsa-miR-187-3p |  |
| hsa-miR-6515-5p | hsa-miR-1914-3p | hsa-miR-378d |  |
| hsa-miR-6072 | hsa-miR-578 | hsa-miR-5681a |  |
| hsa-miR-4704-5p | hsa-miR-1251-3p | hsa-miR-1254 |  |
| hsa-miR-383-3p | hsa-miR-1251-5p | hsa-miR-4796-3p |  |
| hsa-miR-197-5p | hsa-miR-934 | hsa-miR-33a-5p |  |
| hsa-miR-3198 | hsa-miR-4732-5p | hsa-miR-542-3p |  |
| hsa-miR-3121-5p | hsa-miR-4764-5p | hsa-miR-6794-3p |  |
| hsa-miR-142-5p | hsa-miR-513b-5p | hsa-miR-6778-5p |  |

microRNAs with Log 2 (Fold change) < -0.58 and P<0.05 were defined as significantly down-regulated microRNAs

**Supplementary table 3 Significantly up-regulated mRNAs in pcDNA3.1-NEAT1 transfected SNU-182 cells**

| TLDC1 | CBX7 | PIGK | IL17RB | CAPN13 | NFIC | JUNB | EFEMP2 |
| --- | --- | --- | --- | --- | --- | --- | --- |
| VPS53 | ENTHD1 | IFI44L | LPGAT1 | PRKD3 | PIAS4 | TP53I11 | RGS5 |
| SLC43A2 | TNRC6B | PRKACB | GINS4 | PREPL | ELAVL1 | CYB561A3 | EMP2 |
| SMYD4 | UCK2 | SH3GLB1 | HOOK3 | NRXN1 | ZNF426 | SEMA7A | RPL35P5 |
| GALNT6 | TOB2 | C1RL | VCPIP1 | PNPT1 | C19orf66 | SLC14A1 | LIF |
| MMP19 | LRRC1 | DENND5B | SGK3 | PAPOLG | ELAVL3 | MUC1 | RBX1 |
| RBMS2 | CDKL5 | FGD4 | ELOC | UBR5 | FKBP5 | TUBA1C | RPS20P14 |
| INHBC | SH3KBP1 | PPHLN1 | ZC2HC1A | ZNF655 | KCTD20 | RPS5 | RDH5 |
| SLC16A7 | USP9X | TRIP13 | PAG1 | ZKSCAN1 | BTBD9 | TUBB4B | DUSP12 |
| TBC1D30 | TMEM74 | ADCY2 | UQCRB | IFT22 | RPL7L1 | EEF1B2P3 | CLIC1 |
| CPM | EFR3A | ANKH | DCAF4L1 | CUX1 | PAQR8 | HIST1H3B | VPS45 |
| FAM155A | AK3 | CDH12 | GABRA4 | COG5 | RAB23 | ANO3 | ATP6V1C1 |
| AP4S1 | LINGO2 | C1QTNF3 | FRYL | TMEM168 | EYS | GREM2 | MCM7 |
| DTD2 | S100A2 | CCDC125 | SGCB | KCND2 | PGM3 | RARRES3 | TTK |
| KLHL28 | ATP8B2 | F2R | CHIC2 | SLC13A1 | AURKA | HIST1H3I | KIAA0907 |
| L2HGDH | LMNA | ZFYVE16 | CLOCK | CEP41 | PRC1 | PLK1 | STIP1 |
| H2AFV | MEF2D | RASGRF2 | RUFY3 | DGKI | HTR2B | IFITM2 | PARP1 |
| TYW1 | CD1B | POGK | RASSF6 | CREB3L2 | FAM43A | RPLP2 | GOLPH3L |
| TYW1B | GINS2 | GPR161 | LIN54 | ZNF563 | KRT18 | PLK2 | HLTF |
| HIP1 | NOS1AP | CACYBP | WDFY3 | MRI1 | HSPB7 | TOP2A | RGS5 |
| STEAP2 | ZNF264 | GLUL | SESTD1 | IL27RA | IFITM3 | DTL | CHD1L |
| GTPBP10 | PKNOX1 | NMNAT2 | DAPP1 | NWD1 | KIF20A | RELN | MEP1A |
| GATAD1 | ICOSLG | CDK4 | RRAGD | UNC13A | PRC1 | IFI6 | CANT1 |
| RBM48 | ADARB1 | IPO9 | PRDM1 | ZNF101 | SNRPB | B3GAT3 | POLR3C |
| DLK1 | TMEM121B | MDM4 | SOBP | ZNF626 | TAGLN | CLDN11 | TRIP13 |
| PTCD1 | SNAP29 | RASSF5 | TRAF3IP2 | ZFP82 | PSG7 | EMILIN1 | KLHL12 |
| PPP2R3A | LRRC74B | IPCEF1 | NT5DC1 | CELF1 | PTGS1 | NENF | LSM2 |
| SOX14 | HIC2 | GTF2H5 | KIAA0408 | SLC43A1 | SECTM1 | DLGAP5 | CD34 |
| GK5 | TTF2 | TULP4 | SLC2A12 | ZFP91 | ACTA2 | ATP6V0B | LCN2 |
| U2SURP | LCN10 | IQCE | HBS1L | TMEM223 | KRT18 | KRT18P11 | SLC39A1 |
| RNF13 | KLF6 | SP4 | PERP | COL25A1 | SDC4 | IGFBP6 | CLDN15 |
| GPR171 | BEND7 | CREB5 | PHACTR2 | BBS12 | TPM2 | TNFRSF12A | MICB |
| MME | DCLRE1C | WIPF3 | EPM2A | ABHD18 | HLA-B | BUB1B | POGK |
| IFT80 | TRDMT1 | PLEKHA8 | CCDC170 | JADE1 | IFITM1 | RPS10 | FANCI |
| NMD3 | TMEM236 | DDHD1 | ESR1 | NAA15 | TPI1P1 | HLA-C | TRIM24 |
| TTC14 | NEBL | PELI2 | LIN28A | GYPA | TIPARP | LMOD1 | UBE2Q1 |
| KLHL24 | MPP7 | DHRS7 | PHACTR4 | FNIP2 | PTTG1 | LGALS1 | NEDD4L |
| NXT2 | EPC1 | DCAF5 | ZBTB8A | TMEM192 | DDT | SPOCD1 | SRXN1 |
| ACSL4 | CREM | RBM25 | FNDC5 | MFAP3L | SLIRP | SEC61B | HMGB2 |
| AMMECR1 | ZNF33A | VIPAS39 | MEAF6 | JAKMIP2 | SNHG17 | GSTP1 | CCNA2 |
| PAK3 | RET | FOXN3 | AKIRIN1 | FAM114A2 | CHCHD2 | MFSD5 | CLN3 |
| ZBTB33 | VSTM4 | EFCAB11 | OSBPL8 | TMEM97 | HIST1H2BM | MYADM | TUBG1 |
| MCTS1 | ERCC6 | CALM1 | SOCS2 | PHF12 | COX8A | TGFB1I1 | TAGLN2 |
| XIAP | KLF17 | TC2N | VEZT | GOSR1 | CNN1 | PBK | CCT3 |
| AFF2 | TOE1 | MYRIP | ARL1 | MMP28 | PFN1P2 | TPI1 | GMPS |
| GABRA3 | RAB3B | ZNF621 | CMKLR1 | SMIM21 | HAPLN3 | TK1 | TK1 |
| PCDH11Y | NFIA | LRRC2 | VSIG10 | ZNF516 | SNED1 | WDR83OS | FDPS |
| ANKRD54 | KANK4 | PRKAR2A | LDAH | PARD6G | PLIN3 | SEC61G | CCNB1 |
| SUN2 | LEPROT | HEMK1 | UBL4A | POLR2E | ACAN | TUBB3 | BUB1B |
| CENPF | NPLOC4 | SETDB1 | AATF | MTX1 | DAP3 | TYMS | SCGN |
| BARD1 | CKAP2L | SMC4 | KIF4A | HSPB1 | NUAK1 |  |  |

mRNA with Log 2 (Fold change) >0.58 and P<0.05 were defined as significantly up-regulated mRNAs

Supplementary figures


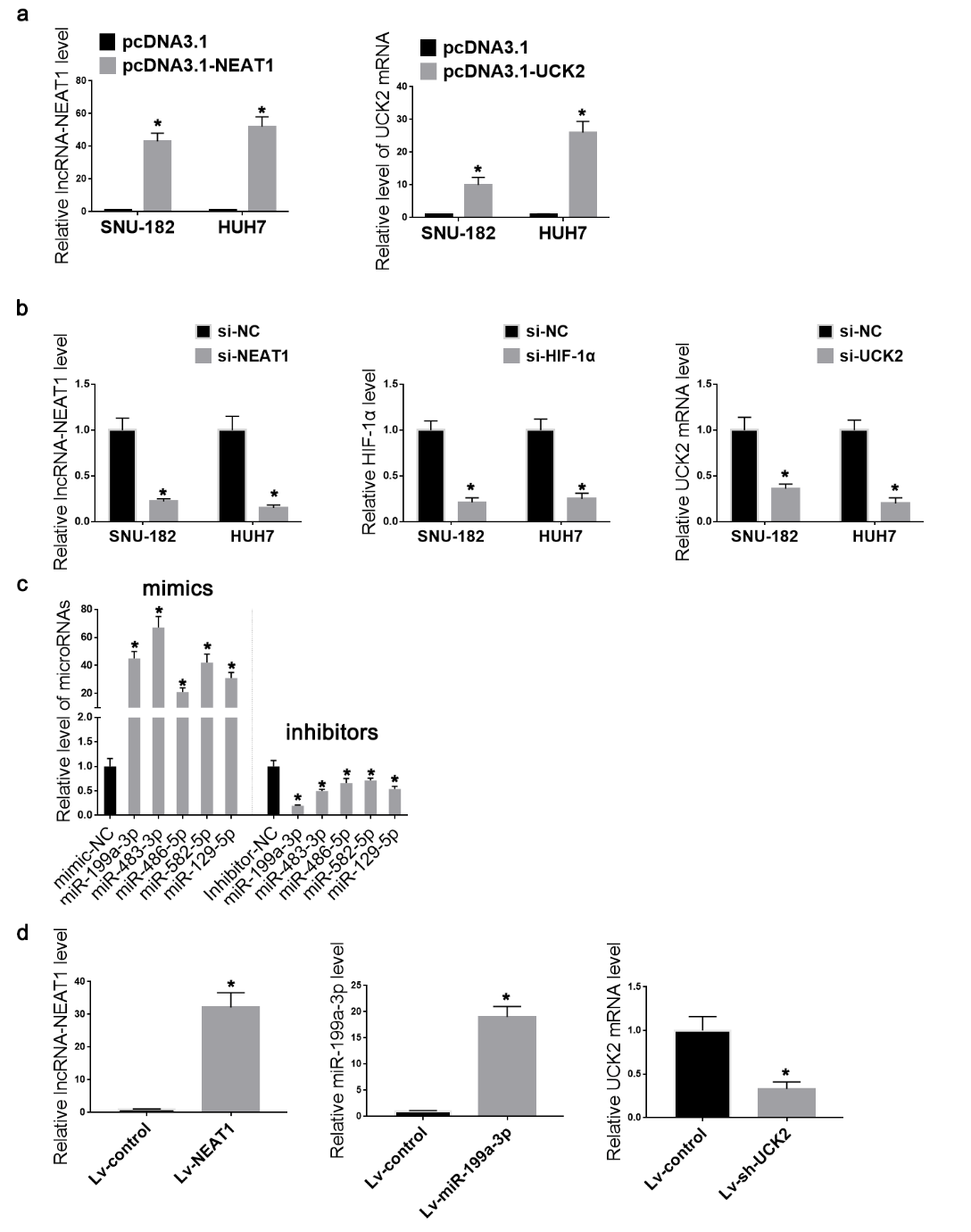


**Supplementary figure 1**

Transfection efficiency of vectors, siRNA, microRNA mimics and microRNA inhibitors were confirmed in SNU-182 cells. **a** the effect of pcDNA3.1 overexpression vector on NEAT1 and UCK2 level. **b** the effect of siRNAs on NEAT1, HIF-1α and UCK2 level. **c** the effect of microRNA mimics and inhibitors on candidate microRNAs. **d** the effect of lentivirus system on NEAT1, microRNA-199a-3p and UCK2 level. **P*<0.05 compared with transfection negative control.


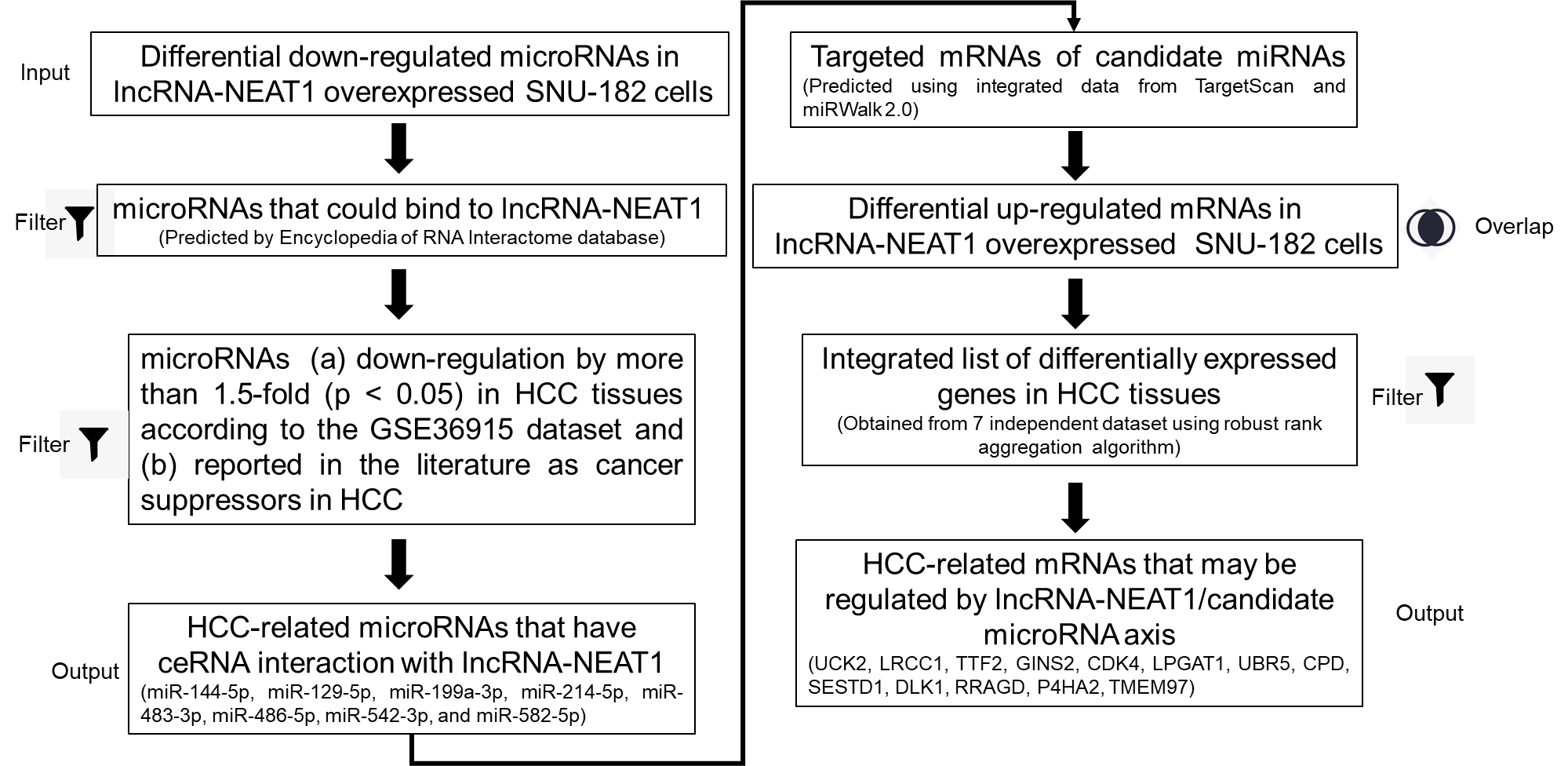


**Supplementary figure 2**

The workflow to build the HCC-related lnc-NEAT1/microRNA/mRNA ceRNA network.


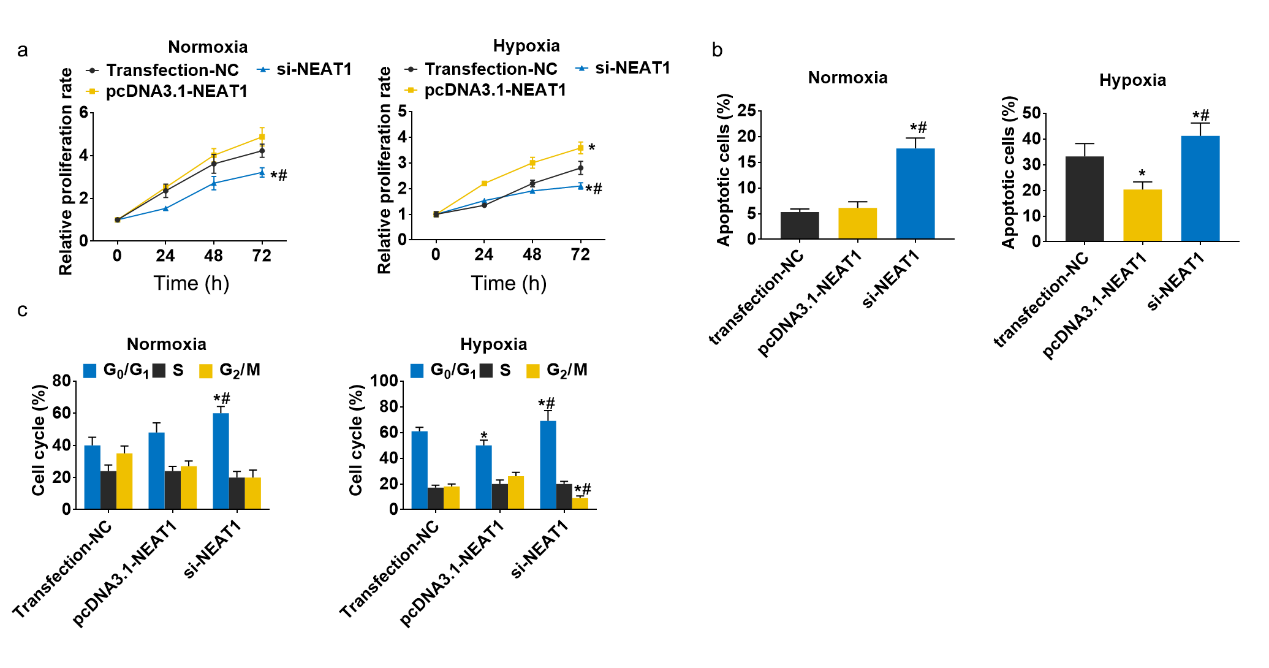


**Supplementary figure 3**

LncRNA-NEAT1 sustained the growth of HUH7 cells under hypoxic conditions. **a** The expression of lncRNA-NEAT1 was altered by transfection with the pcDNA3.1-NEAT1 vector or siRNA-NEAT1. After transfection for 24 h, cells were cultured under hypoxic or normoxic conditions. Cell viability was detected using CCK-8 assay and used to estimate proliferation. **b**  and **c** The roles of lncRNA-NEAT1 on apoptosis and the cell cycle of HUH7 cells under hypoxic and normoxic conditions were assessed by flow cytometry. **P*<0.05 compared with transfection negative control. ^#^*P*<0.05 compared with pcDNA3.1-NEAT1.


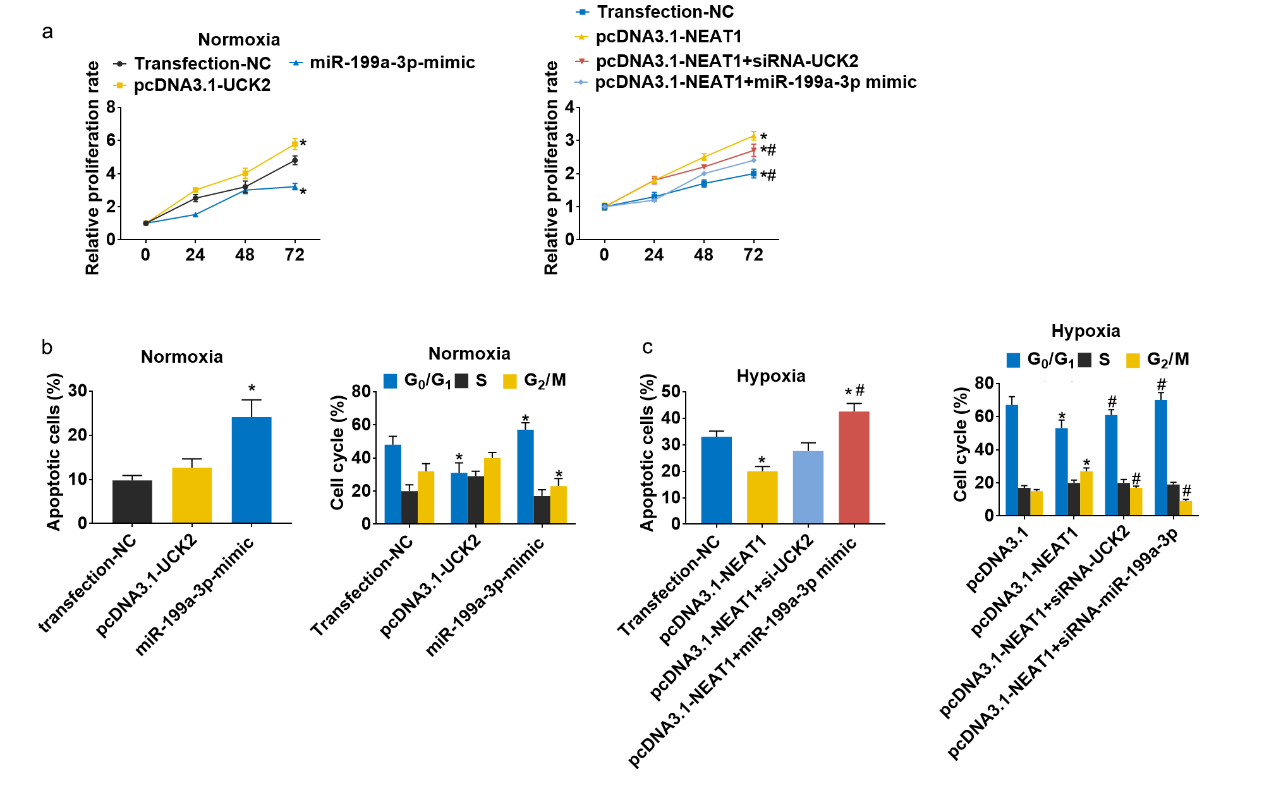


**Supplementary figure 4**

LncRNA-NEAT1 sustains the growth of HUH7 cells under hypoxic conditions by regulating miR-199a-3p/UCK2. **a** the expression levels of miR-199a-3p and UCK2 in HUH7 were up-regulated by transfection. On the other hand, miR-199a-3p or siRNA-UCK2 were co-transfected with pcDNA3.1-NEAT1 in HUH7 cells under hypoxia. The cell proliferation changes were determined with the CCK-8 assay. **b** and **c** the effects of miR-199a-3p and UCK2 on apoptosis and the cycle of HUH7 cells were determined by flow cytometry. **P*<0.05 compared with transfection negative control. ^#^*P*<0.05 compared with pcDNA3.1-NEAT1.
